# Supplementary material for: Translating metabolomic evidence gathered from an animal model to a real human scenario: the post-mortem interval issue
Source: Metabolomics. 2025 Aug 21;21(5):125. doi: 10.1007/s11306-025-02321-4 (PMC12370792; doi:10.1007/s11306-025-02321-4)
Supplement: Supplementary file 1 — Supplementary Material 1 [file 11306_2025_2321_MOESM1_ESM.docx]

**Supplementary Table 1.** List of AH metabolites quantified using the Chenomx Profiler tool.

| **Species** | **Compound** | **PubChem**  **(CID)** | **Species** | **Compound** | **PubChem**  **(CID)** |
| --- | --- | --- | --- | --- | --- |
| A, H | 2-Hydroxybutyrate | 11266 | H | Ethanolamine | 700 |
| A, H | 3-Hydroxybutyrate | 441 | H | Glucose | 5793 |
| A, H | 3-Hydroxyisobutyrate | 87 | H | 2-Hydroxyisovalerate | 99823 |
| A, H | Acetate | 175 |  |  |  |
| A, H | Acetone | 180 |  |  |  |
| A, H | Alanine | 5950 |  |  |  |
| A, H | Arginine | 6322 |  |  |  |
| A, H | Ascorbate | 54670067 |  |  |  |
| A, H | Asparagine | 6267 |  |  |  |
| A, H | Aspartate | 5960 |  |  |  |
| A, H | Betaine | 247 |  |  |  |
| A, H | Choline | 305 |  |  |  |
| A, H | Citrate | 311 |  |  |  |
| A, H | Creatine | 586 |  |  |  |
| A, H | Creatinine | 588 |  |  |  |
| A, H | Dimethylsulfone | 6213 |  |  |  |
| A, H | Dimethylamine | 674 |  |  |  |
| A, H | Formate | 283 |  |  |  |
| A, H | Glutamate | 33032 |  |  |  |
| A, H | Glutamine | 5961 |  |  |  |
| A, H | Glycerol | 753 |  |  |  |
| A, H | Glycine | 750 |  |  |  |
| A, H | Histidine | 6274 |  |  |  |
| A, H | Hypoxanthine | 135398638 |  |  |  |
| A, H | Inosine | 135398641 |  |  |  |
| A, H | Isoleucine | 6306 |  |  |  |
| A, H | Lactate | 612 |  |  |  |
| A, H | Leucine | 6106 |  |  |  |
| A, H | Lysine | 5962 |  |  |  |
| A, H | Methanol | 887 |  |  |  |
| A, H | Methionine | 6137 |  |  |  |
| A, H | Ornithine | 6262 |  |  |  |
| A, H | Phenylalanine | 6140 |  |  |  |
| A, H | Serine | 5951 |  |  |  |
| A, H | Succinate | 160419 |  |  |  |
| A, H | Taurine | 1123 |  |  |  |
| A, H | Tryptophan | 6305 |  |  |  |
| A, H | Tyrosine | 6057 |  |  |  |
| A, H | Uracil | 1174 |  |  |  |
| A, H | Valine | 6287 |  |  |  |
| A, H | myo-Inositol | 892 |  |  |  |
| A, H | sn-Glycero-3-phosphocholine | 657272 |  |  |  |
| A, H | τ-Methylhistidine | 64969 |  |  |  |
